# Polyunsaturated Fatty Acid Intake during Complementary Feeding and Neurodevelopmental Outcome in Very Low Birth Weight Infants

**DOI:** 10.3390/nu15143141

**Published:** 2023-07-14

**Authors:** Melanie Gsoellpointner, Margarita Thanhaeuser, Fabian Eibensteiner, Robin Ristl, Bernd Jilma, Renate Fuiko, Sophia Brandstetter, Angelika Berger, Nadja Haiden

**Affiliations:** 1 Department of Clinical Pharmacology, Medical University of Vienna, 1090 Vienna, Austria; melanie.gsoellpointner@meduniwien.ac.at (M.G.); bernd.jilma@meduniwien.ac.at (B.J.); 2 Department of Pediatrics and Adolescent Medicine, Comprehensive Center for Pediatrics, Medical University of Vienna, 1090 Vienna, Austria; margarita.thanhaeuser@meduniwien.ac.at (M.T.); fabian.eibensteiner@meduniwien.ac.at (F.E.); renate.fuiko@meduniwien.ac.at (R.F.); sophia.brandstetter@meduniwien.ac.at (S.B.); angelika.berger@meduniwien.ac.at (A.B.); 3 Center for Medical Data Science, Medical University of Vienna, 1090 Vienna, Austria; robin.ristl@meduniwien.ac.at; 4 Department of Neonatology, Kepler University Hospital, Johannes Kepler University, 4020 Linz, Austria

**Keywords:** polyunsaturated fatty acids, docosahexaenoic acid, arachidonic acid, neurodevelopment, preterm infants

## Abstract

Polyunsaturated fatty acids (PUFAs) are vital for brain development, yet limited knowledge exists regarding PUFA intake during complementary feeding (CF) and its impact on neurodevelopmental outcomes in very low birth weight (VLBW) infants. This secondary analysis of a randomized intervention trial, aimed to investigate the association between dietary intake of total PUFAs, arachidonic acid (AA), and docosahexaenoic acid (DHA) during CF and neurodevelopmental outcomes at 12 and 24 months of corrected age (CA). Dietary intakes were assessed using monthly 3 day dietary protocols from 3 to 12 months CA. Neurodevelopmental outcome was evaluated using the Bayley Scales of Infant Development-III. Among the 177 randomized patients, PUFA intake and neurodevelopmental outcomes were evaluated in 140 (79%) infants. Higher total PUFA and DHA intakes significantly correlated with improved cognitive and motor function at 12 months CA, while increased AA intake notably enhanced motor scores at 12 months CA. However, median dietary intakes of AA and DHA (AA: 53.50–84.25 mg/d; DHA: 51.47–76.23 mg/d) fell short of recommended levels (AA: 140 mg/d; DHA: 100 mg/d) at any of the investigated timepoints. These findings emphasize the need to enhance total PUFA, DHA and AA intakes during CF, ensuring adherence to guidelines and unlocking the potential to improve neurodevelopmental outcomes in VLBW infants.

## 1. Introduction

Polyunsaturated fatty acids (PUFAs), specifically the *n*-3 fatty acid docosahexaenoic acid (DHA) and the *n*-6 fatty acid arachidonic acid (AA), are essential nutrients that significantly contribute to the brain development of preterm infants during both the perinatal and postnatal period [1]. The accretion of fatty acids in adipose tissue and in the brain primarily takes place during the third trimester of pregnancy, which puts premature infants at higher risk of PUFA deficiency due to the early termination of pregnancy [2,3]. Consequently, the postnatal period becomes a critical phase for ensuring an adequate PUFA intake. DHA and AA can be obtained directly through the infants’ diet or synthesized from essential precursors, namely alpha-linolenic acid (ALA) and linoleic acid (LA) [4]. However, the capacity to synthesize sufficient amounts from these precursors might be inadequate to meet the nutritional requirements during early infancy [5]. The topic of PUFA supplementation during the early postnatal phase is still under discussion. Nonetheless, there is a general consensus regarding the importance of providing adequate PUFA supply as deficiency of these nutrients can negatively impact cell signaling and lead to impaired behavior, learning and cognitive abilities [6]. Although there is an increasing body of evidence suggesting the potential positive role of PUFAs in the brain and neurodevelopment [7,8], there is currently a lack of data on dietary PUFA intake during complementary feeding (CF) and its association with neurodevelopmental outcome in very low birth weight (VLBW) infants. Up to now, only one study has examined the association of PUFA intake and neurological development during CF in healthy full term infants. The PINGU (Polyunsaturated fatty acids in child nutrition—a German multimodal optimization study) trial revealed that regular fish consumption, as part of a structured infant diet enhanced DHA and eicosatetraenoic acid (EPA) serum levels [9] but did not impact cognitive development at 10 months of age [10]. However, preterm infants might be more likely to benefit from higher PUFA intakes compared to term infants as they are at a greater risk of deficiency [3].

Therefore, this secondary analysis of the PIES (Preterm Infants on Early Solid Foods) study aims to investigate the association between PUFA intake during the CF period and neurodevelopmental outcome at 12 and 24 months of corrected age (CA) in VLBW infants. Additionally, this study provides data on current PUFA intakes during the first year CA and whether the recommended dietary guidelines are being met.

## 2. Materials and Methods

### 2.1. Study Design and Subgroup

This is a secondary analysis of nutritional data collected during a prospective, randomized, two arm intervention trial and the association with neurodevelopmental outcome in infants born with a birth weight <1500 g. The initial study was designed to investigate the introduction of CF in VLBW infants at two different timepoints. Infants were either introduced to solids between the 10th and 12th week CA (early group) or between the 16th and 18th week CA (late group) and fed a standardized feeding concept throughout the first year CA. Exclusion criteria were any diseases that might affect stable growth (e.g., Hirschsprung disease, inflammatory bowel syndrome, necrotizing enterocolitis with short bowel syndrome, any chromosomal aberration, congenital heart disease, major congenital birth defect). The study was conducted from October 2013 until February 2020 in the outpatient clinic of the Division of Neonatology, Department of Pediatrics, Medical University of Vienna and approved by the ethics committee of the Medical University of Vienna (EK: 1744/2012). The detailed study design was described previously [11] and the study protocol can be accessed on ClinicalTrials.gov (NCT01809548).

### 2.2. Standardized Feeding Concept and Dietary Intake Analysis

Study participants had to follow a standardized feeding concept that was ready-to-use, commercially available baby jar food provided for free by Nestle^®^ company (Vienna, Austria). The age-dependent step-up feeding concept started with pureed fruits and vegetables in the scoop familiarization phase and was extended to include meat, grains, fish and milk products in the later phases of CF. Throughout the study, parents were required to maintain detailed 3-day dietary records to ensure at least 80% adherence to the study’s food guidelines [12,13]. All dietary intakes, including milk intake, had to be listed monthly on three consecutive days including one weekend day, from 3 to 12 months CA (M3–M12). To ensure accurate nutrient analysis, detailed information on the used formula had to be documented. Recipes of all infant formula were requested by the distinct manufacturers and changes in formulations were considered for calculation. In breastfed infants, the exact milk intake was unknown, hence, estimated average values of consumed mother´s milk, published by Dewey KG et al. [14] were used for calculation. Mean dietary intakes of each participant were calculated from the 3-day dietary records by a nutritionist using nut.s nutritional.software (Vienna, Austria) based on the German Nutrient Data Base and the Austrian nutrient table (Version II.3.1).

Within this paper, we analyzed single component dietary intake (DHA, AA) as well as total PUFA intake, defined as all fatty acids with a minimum of 2 double bonds. PUFAs are categorized into 2 main families: omega-3 (*n*-3) and omega-6 (*n*-6) fatty acids as given in Table 1 [15]. In addition to the main fatty acids, several other PUFAs that are present only in small quantities count towards the total PUFA intake; however, they are not mentioned separately within this paper. For primary and secondary outcome analysis, mean dietary intakes of total PUFAs, DHA and AA per day throughout the CF period were calculated from all available records per participant. We further provide intake data of PUFA, DHA, AA, ALA, LA and LA/ALA ratio monthly from M3 to M12 and compared them with the current dietary guidelines. Additional methodological details regarding the feeding concept and dietary intakes can be found in the initial dietary intake report of this study [16].

### 2.3. Neurodevelopmental Assessment

Neurodevelopmental assessment was conducted using the Bayley Scales of Infant and Toddler Development, third edition (Bayley-III; Harcourt Assessment, San Antonio, TX, USA, 2006) [17] at 12 and 24 months CA as part of clinical routine care. The Bayley-III is an individually administered instrument to assess the development of infants and young children between 1 and 42 months. It consists for three scales, the Cognitive Scale, the Language Scale, including receptive and expressive communication, and the Motor Scale, including fine and gross motor function. A Social-Emotional Scale and Adaptive Behavior Scale can be completed by parents or primary caregivers. However, within this analysis, only the domains cognition, language and motor were used for outcome assessment. The Bayley-III Score ranges are 55–145, 46–154 and 47–153 in cognition, motor and language, respectively. A score of 100 in any composite score defines the average performance of a given age group and scores between 85–115 are 1 standard deviation below and above the mean. Two certified clinical psychologists performed and scored the tests. Scaled scores were calculated for each test and the scores were further converted into cognitive, language and motor composite scores. In this secondary outcome analysis, results were calculated using the Bayley-III US norms.

### 2.4. Outcome Parameters

The primary outcome of this study was to assess the effect of dietary mean total PUFA intake during the first year of life on neurodevelopmental outcome (cognition, motor, language) at 12 months CA. One secondary outcome was the association between dietary mean total PUFA intake and cognition, motor and language development at 24 months CA. Another was the association of mean dietary DHA and AA intake during the first year of life and neurodevelopmental outcome at 12 and 24 months of life CA. In addition, we conducted a subgroup analysis to investigate the association of PUFAs with developmental neurological outcomes between sexes, which can be found in the Supplementary Materials.

### 2.5. Statistical Analysis

Statistical analysis was performed on the intention-to-treat analysis set (all randomized patients were included), with the exclusion of subjects that were lost for follow-up, moved, no dietary records or withdrawn informed consent. As the number of participants in this analysis was smaller compared to the initial randomized dataset, baseline characteristics and dietary intakes are reported from all participants valid for the analysis of the defined primary outcome, i.e., all subjects in which at least one 3-day dietary record and Bayley-III outcome assessment at 12 months CA was available. In the following, we defined this dataset as “PUFA subgroup”. For ordinal and nominal variables, absolute and relative frequencies are reported and continuous variables are presented using median and interquartile range (IQR). The primary outcome was assessed using linear mixed-effects models with PUFA intake, gestational age at birth, intervention group, sex, highest parental education, nutrition at discharge (breastfed, formula, mixed (breastmilk and formula), and intraventricular hemorrhage (Grades III + IV)) as fixed effects, and a random intercept to adjust for possible correlation between siblings of multiple births. Model estimates and 95% confidence intervals are reported. Secondary outcomes were assessed in the same manner as the primary outcome. As an additional analysis, *p*-values referring to primary and secondary outcome analyses of the same nutrient were adjusted using the Bonferroni–Holm method. Both adjusted and unadjusted *p*-values are displayed. All tests were two-sided at an alpha of 0.05. Statistical analysis was conducted using statistical computing software R (R Foundation for Statistical Computing, Vienna, Austria, Version 3.5 or higher).

## 3. Results

### 3.1. Screening and Participants

**Figure 1:**
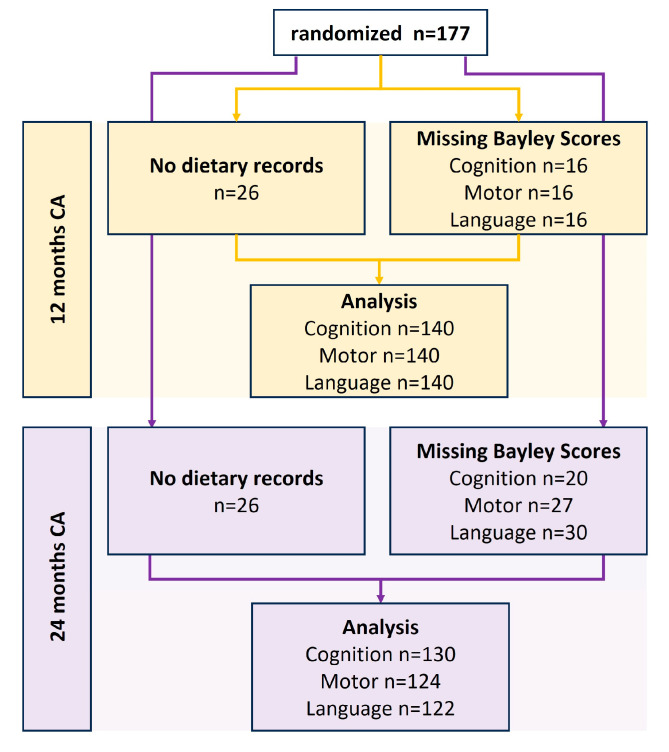
Flow chart of study profile.

Out of the 177 patients that were randomized, neurodevelopmental outcome data at 12 months CA and at least one dietary record were available for analysis in 140 patients, accounting for 79% of the total sample size (Figure 1). A total of 1100 valid 3-day dietary records were included in this analysis.

### 3.2. Baseline Characteristics and Neonatal Morbidity

Neonatal and obstetric parameters are shown in Table 2. The baseline characteristics and neonatal outcome parameters are reported specifically for this PUFA subgroup, which was derived from the initial randomized patient set. Upon comparison, no differences were found between baseline characteristics of the PUFA and the initial intention-to-treat dataset. Detailed information can be found in Supplementary Table S1. A comparative analysis between the PUFA subgroup and a subset of infants lost to follow-up (*n* = 37) revealed notable disparities at baseline, including variances in the prevalence of anemia, maternal age at birth, and complete administration of perinatal steroids. Specifically, the PUFA subgroup exhibited lower rates of anemia (PUFA subgroup: 7%, Lost to follow-up: 22%, *p* = 0.03), as well as reduced exposure to perinatal steroids (PUFA subgroup: 55%; Lost to follow-up: 73%, *p* = 0.01). Additionally, mothers belonging to the PUFA subgroup exhibited a statistically significant higher mean age compared to the lost to follow-up group (PUFA subgroup: 33 (30–37); Lost to follow-up: 30 (24–33), *p* = 0.01; Supplementary Table S2). While these baseline characteristics exhibited differences, we posit that this baseline heterogeneity is unlikely to impact the outcomes of the analysis, as higher perinatal steroid exposure and anemia may indicate lower neurodevelopmental outcomes in the lost to follow-up group rather than the PUFA subgroup (Supplementary Table S2).

### 3.3. Dietary Total PUFA, DHA and Intake during the First Year of Life

Although individual mean intakes were considered for primary and secondary outcome analyses, we herein report median dietary intakes and interquartile range across the first year of life of the PUFA subgroup. The median dietary intakes of total PUFAs, DHA and AA throughout the first year of life were 4.55 g/d (IQR: 3.69–5.41 g/d), 60.08 mg/d (IQR: 34.72–85.43 mg/d) and 64.39 mg/d (IQR: 33.45–90.31 mg/d), respectively.

### 3.4. Fatty Acid Intake from 3 to 12 Months CA and Comparison with Current Dietary Intake Recommendations

**Figure 2:**
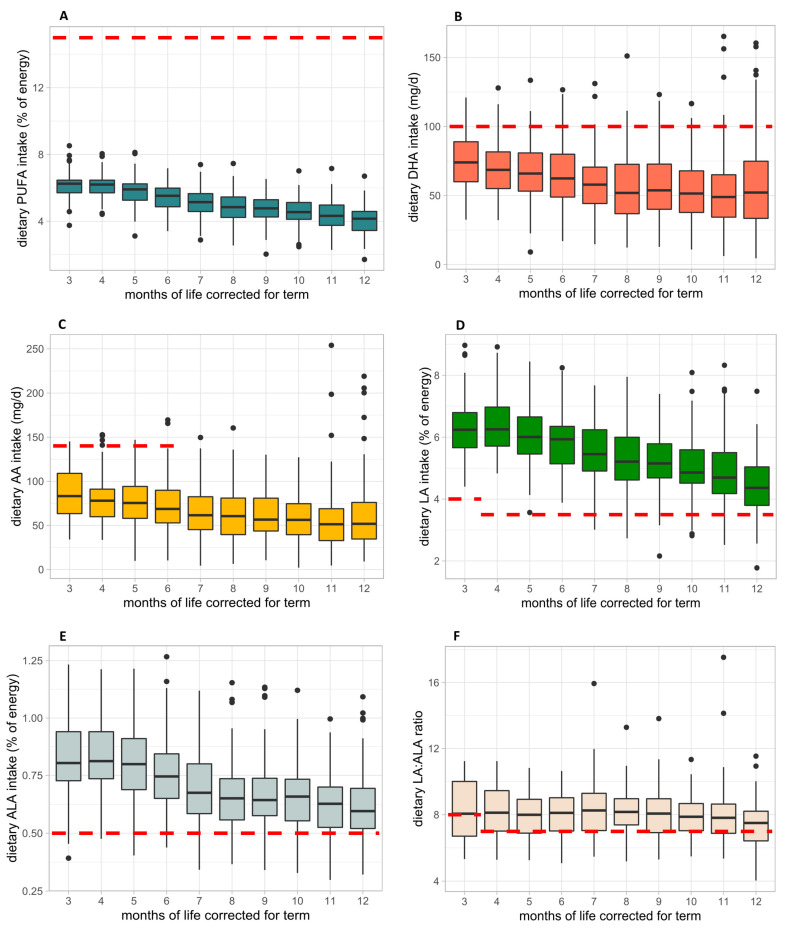
Dietary fatty acid intake from 3 to 12 months of life CA in comparison with dietary intake recommendations. (**A**) Polyunsaturated fatty acid (PUFA) in % of energy, (**B**) docosahexaenoic acid (DHA) in mg/d, (**C**) arachidonic acid (AA) in mg/d, (**D**) linoleic acid (LA) in % of energy, (**E**) alpha-linolenic acid (ALA) in % of energy, and (**F**) LA/ALA ratio. The red line represents the dietary intake recommendation for the distinct nutrient.

Median monthly dietary PUFA intake (% of energy) from M3 to M12 is shown in Figure 2A. Median PUFA intake decreased over time, with levels ranging from 6.25% (IQR:5.43–7.07%) at M3 to 4.11% (IQR: 2.97–5.25%) at M12; hence, they were within the recommendations (<15% from total energy/d) [18] at all of the investigated time points. Dietary DHA (mg/d) intake decreased from 76.23 mg/d (IQR: 43.35–107.11 mg/d) at M3 to 51.47 mg/d (IQR: 13.01–89.93 mg/d) at M12 (Figure 2B). Dietary recommendations for the daily intake of DHA were met (100 mg/d) only at M3 [19]. From birth to 6 months of age, it is recommended to provide a daily intake of 140 mg AA [20]. Median AA intake was below these recommendations. Dietary AA (mg/d) intake was highest at M3 (median: 84.25 mg/d; IQR: 39.02–129.47 mg/d) and decreased to 53.50 mg/d (IQR: 12.43–94.57 mg/d) at M12 (Figure 2C). Dietary LA intakes were above the recommended daily intake (0–3 months: 4.0% of energy; 4–12 months: 3.5% of energy) [21] from M3 to M12 (Figure 2D). Median LA intake (% of energy) was 6.26% (IQR: 5.14–7.38%) at M3 and decreased to 4.35% (IQR: 3.13–5.57%) at M12. Similar to the daily intake of LA, intake recommendations for ALA are based on percentage of total energy, namely a minimum dietary requirement of 0.5% of total energy from 0 to 12 months of life [21]. Median daily ALA intake met these recommendations at any of the investigated months with proportional intakes of 0.80% (IQR: 0.57–1.03%) at M3 and 0.59% (IQR: 0.44–0.74%) at M12 (Figure 2E). The ratio of LA/ALA was close to the recommended values (0–3 months: 8:1; 4–12 months: 7:1) [21] throughout the first year of life (Figure 2F) with a ratio of 8.1:1 (IQR: 4.8–11.4:1) at M3 and 7.5:1 (IQR: 5.8–9.2:1) at M12.

### 3.5. Bayley-III: Cognition, Motor, Language

**Figure 3:**
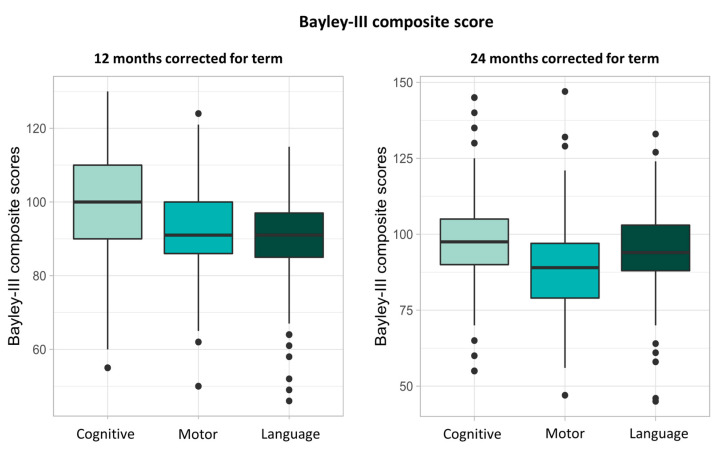
Bayley-III composite scores at 12 and 24 months corrected for term.

Results of Bayley-III assessment are shown in Figure 3. Median cognitive composite score at 12 months CA was 100 (IQR: 80–120, *n* = 140) and 97.5 (IQR: 82.5–112.5, *n* = 130) at 24 CA. Median motor composite score at 12 months CA increased from 91 (IQR: 79–103, *n* = 140) to 94 (IQR: 79–109, *n* = 124) at 24 months CA. At 12 months CA, median language score was 91 (IQR: 77–105, *n* = 140) and decreased to 89 (IQR: 71–106, *n* = 122) at 24 months CA.

### 3.6. Total PUFA Intake and Neurological Development

Results of the primary outcome are shown in Table 3. The amount of PUFA intake during the first year of life was positively associated with better cognition at 12 months CA (*p* = 0.0005). The estimated slope of 7.32 (see Table 3) can be interpreted as an average value calculated over the available data. This means that in a population with comparable distribution of PUFA intake as in the study sample, a difference in 1 g/d PUFA intake between two subjects would yield an average corresponding to a difference of 7.32 points in the cognition score. After adjustment for multiple testing, the primary outcome remained significant (*p* = 0.003). However, no association was found for PUFA intake during CF and cognitive composite score at 24 months CA. Infants with a higher PUFA intake during the first year had significantly better motor function at 12 months CA (*p* = 0.004), which also remained significant after adjustment for multiple testing (*p* = 0.01). Although there was a trend towards better motor function at 24 months CA, the results did not reach statistical significance (*p* = 0.06). No association was found for PUFA intake and language development at 12 and 24 months CA.

To evaluate whether the observed relation between total PUFA intake and neurodevelopmental outcome resulted from DHA or AA intake, we further evaluated the association between DHA and AA intake during the first year of life and neurodevelopment at 12 and 24 months CA.

### 3.7. DHA Intake and Neurological Development

Higher dietary DHA intake throughout the first year of life was associated with significantly better cognitive function at 12 months CA (*p* = 0.04, *p*-adjusted = 0.13). Furthermore, DHA intake was significantly associated with improved motor function at 12 months CA (*p* = 0.002, *p*-adjusted = 0.01). Cognition and motor score were not significant at 24 months CA. No associations were identified for DHA intake and language score at 12 and 24 months CA (Table 4).

### 3.8. AA Intake and Neurological Development

Infants who had a higher dietary AA intake throughout the first year of life had significantly better motor scores at 12 months CA (*p* = 0.004, *p*-adjusted = 0.03) but not at 24 months CA. No associations were found for AA intake on cognition and language score at 12 and 24 months CA (Table 5).

## 4. Discussion

### 4.1. Dietary Intake of Total PUFAs and Neurodevelopmental Outcome

This is a secondary outcome analysis of a randomized intervention trial investigating the dietary intake of total PUFAs, DHA and AA during the first year of life and its associations with neurodevelopmental outcome. To the best of our knowledge, there are no published data on dietary intakes of PUFAs during the complementary feeding period and its association with neurodevelopmental outcome in VLBW infants. In this study, we were able to demonstrate that a higher total PUFA intake during the first year of life was significantly associated with better developmental scores, particularly for cognitive and motor scores at 12 months CA. Infants who had higher intakes of total PUFAs also exhibited higher motor and cognition scores at 24 months CA (effect size: 2.74 and 3.37), although the association was not statistically significant. We speculate that the lack of statistical significance may be due to the discontinuation of the standardized feeding concept after the first year of life. Additionally, the subgroup analysis examining the association between PUFA intake and neurodevelopmental outcome across sexes demonstrated that higher intakes of total PUFAs, AA and DHA had a significantly greater positive impact on neurodevelopmental outcomes in male subjects compared to their female counterparts. Therefore, it appears that the intake of PUFAs has sex-specific effects on neurodevelopmental outcomes.

Although there are no recommendations on the minimal absolute daily intake, PUFA intake is recommended to be less than 15% of energy for infants up to 24 months of life [18]. In the present study, the median PUFA intake decreased from 6.25 to 4.11% of energy throughout the first year of life with some individual levels slightly exceeding 8% of energy (Figure 2A). It is possible that a higher PUFA intake within the safe range (<15%) could be advantageous in realizing the full potential of PUFA intake for long-lasting positive effects on neurodevelopment. However, this assumption remains hypothetical as the statistical analysis of our study does not provide conclusive evidence of linearity in our results. In addition, there is currently a lack of studies on total PUFA intake during CF and its impact on neurological development in VLBW infants. Further randomized controlled trials with higher total PUFA intakes in preterm infants are needed to validate this hypothesis.

### 4.2. Dietary Intakes of DHA and AA and Neurodevelopmental Outcome

In the present study, we found that higher DHA and AA intakes during the first year of life were significantly associated with better neurodevelopmental outcomes at 12 months CA, but not at 24 months CA. So far, only one study investigated the association between DHA intake during CF and neurodevelopmental outcome; however, that study was in healthy full term infants. In the PINGU (Polyunsaturated fatty acids in child nutrition—a German multimodal optimization study) trial infants received ready-made complementary foods with either ALA-rich rapeseed oil (IG-R), salmon (two times per week) to provide preformed DHA (IG-F) or LA-rich corn oil (control group). Infants in the IG-F group showed enhanced DHA and EPA serum levels [9] compared to the control group; however, cognitive development at 10 months of age did not differ between the groups [10]. The study authors suggested that the lack of measurable effects on neurodevelopment might be attributed to “brain growth spurts” that could hinder the detection of small effects from dietary intervention. The timing and duration of the intervention period were also mentioned as possible explanations for the lack of effects [10]. Preterm infants may be more likely to benefit from higher PUFA intakes, especially DHA, compared to term infants due to their increased risk of deficiency [3]. Previous research has shown that infants born extremely premature may benefit the most from higher PUFA intakes [22]. In a multicenter randomized-placebo controlled trial conducted by Makrides et al., infants with a birth weight of less than 1250 g and a higher DHA intake (approximately 1% of total fatty acids) had a better mental development index (MDI) compared to controls, although this effect was only observed in the unadjusted analysis [23]. This study also demonstrated that higher DHA supply reduced the frequency of mild mental delay by approximately 45% in those born with a birth weight of less than 1250 g. Although the findings of our study should be interpreted cautiously because the study was not powered to detect a significant association between DHA and AA intake and neurological outcome, the results are consistent with previous literature suggesting a potential benefit in those with the highest requirements and the highest risk of deficiency.

In the present study, median DHA intake was very low and current recommendations (100 mg/d) [19] were not met at any of the investigated timepoints (Figure 2B). Based on the average intake throughout the first year, calculated as mean of all monthly protocols per participant, only 3.6% of infants met the recommended DHA of 100 mg/d. A comparison of AA intake and current recommendations was only possible until 6 months CA, as there are no specific recommendations for the second half of the first year of life. AA intake was below the recommended intake of 140 mg/d [20] until 6 months CA and steadily decreased reaching its lowest levels at 12 months CA (53.50 mg/d) (Figure 2C). This is consistent with previous studies showing that with the introduction of CF and the gradual decrease of human milk and/or PUFA-enriched formula, intakes of DHA and AA substantially decrease in the second half of the first year of life [24,25]. Although median dietary intakes of AA and DHA were generally low, we could still show that infants with higher intakes had better neurodevelopmental outcome at 1 year CA. Improving AA and DHA intakes and aiming toward a guideline diet for PUFA intake should, therefore, be considered as viable goals to improve neurodevelopmental outcome in this vulnerable population.

### 4.3. Dietary Intakes of LA, ALA and LA/ALA Ratio

We further conducted a detailed analysis of the dietary LA and ALA intake, as they are essential fatty acids that cannot be synthetized in the human body and must, therefore, be obtained through the diet. Moreover, ALA and AA are precursors for DHA and AA. It is well-established that not only fatty acid quantity, but also the ratio of LA and ALA plays a crucial role in determining the nutritional adequacy of PUFAs [26]. The German Nutrition Society (DGE) generally recommends a daily intake of 0.5% energy as ALA during the first year of life, and 4.0 and 3.5% energy as LA from 0 to 3 months and 4 to 12 months, respectively. Consequently, the recommended dietary LA/ALA ratio is 8:1 until 3 months of age and 7:1 from 4 to 12 months [21]. Previous studies reported negative associations between a high LA/ALA ratio and cognition [27,28]. Bernard et al. found that high levels of LA in human milk were negatively associated with motor and cognitive scores [29]. Furthermore, a high maternal LA/ALA ratio was associated with poorer neurodevelopmental outcome at 9 months and 3 years, whereas a lower ratio was linked to fewer emotional problems during childhood [30,31]. The DINO trial (Dortmund Intervention Trial for Optimization of Infant Nutrition) was able to show that higher intakes of ALA and a lower ratio of LA/ALA (10.7 versus 14.8) during the CF period increased plasma proportions of total *n*-3 fatty acids and *n*-3 PUFA at 10 months of life [32]. However, it is important to note that this study was conducted in healthy full term infants and did not provide data on neurodevelopmental outcome. While we cannot draw any conclusions about the association of the LA/ALA ratio and neurodevelopmental outcome from this study, the overall positive effect of total PUFAs on cognition and motor development may be partially attributed to the LA/ALA ratio, which was closely aligned with the recommended values. Further studies on PUFA quantity and quality during CF with respect to neurodevelopmental outcome are much needed to improve neurological development in VLBW infants.

### 4.4. How to Improve Dietary PUFA Intake to Meet a Guideline Diet?

Based on the findings of this study, it is desirable to increase the dietary intake of PUFAs in order to meet nutritional requirements and promote better neurological development in VLBW infants during their first year of life. Breast milk is considered the optimal choice for infant feeding and naturally contains both DHA and AA [33]. However, due to the decrease in breast milk volume over time, additional dietary sources of PUFAs from non-human milk become necessary to meet the recommended intakes. ALA found in flaxseed, canola, soybean and walnut is the main source of *n*-3 PUFAs. It is important to note that the conversion rate from ALA to DHA is very low in infants, thus, it is recommended to incorporate marine sources rich in DHA to meet the recommended nutritional supply of *n*-3 fatty acids [5]. DHA is mainly found in salmon, sardines, tuna, trout and to a lesser extent in chicken [26]. In order to meet the nutritional recommendation of DHA, the German Society of Pediatric and Adolescent Medicine recommends one to two fish meals per week, with each consisting of 20–30 g of fatty fish [34]. AA is particularly abundant in eggs, meat, fish and seafood [35,36,37]. Increasing the consumption of these foods with a particular emphasis on at least one fish meal per week may contribute to a higher total PUFA intake, including DHA and AA, and therefore potentially improving neurodevelopmental outcome in VLBW infants. Additionally, rapeseed oil, with its favorable LA/ALA ratio of 2:1, can be beneficial for achieving a balanced ratio during CF and can be used in the preparation of homemade complementary foods [34]. Furthermore, various dietary interventions have been explored as potential means to enhance the neurodevelopmental outcomes of preterm infants. A noteworthy example is a study published in 2020, which demonstrated the potential benefits of employing a ketogenic diet as an alternative therapeutic approach for managing drug-resistant seizures, as well as neurological disorders, neurodevelopmental disorders and mood disturbances [38]. There is a strong need for future studies aimed at investigating the efficacy of dietary interventions in promoting neurological development. Additionally, it is crucial to explore the potential impact of these interventions on emotional well-being. Such studies would provide valuable insights into the relationship between diet and both neurological and emotional aspects, contributing to a more comprehensive understanding of the potential benefits of dietary approaches in promoting optimal neurodevelopmental outcomes.

### 4.5. Study Strength and Limitations

This study has several notable strengths. Firstly, the implementation of a standardized CF concept ensured consistency and accuracy in assessing PUFA intake, which allowed for precise calculations during the CF period. Additionally, the inclusion of monthly dietary records throughout the first year of life provided comprehensive and continuous data on dietary intake. However, it is important to acknowledge the limitations of this study. As a secondary outcome analysis of a randomized controlled trial, the study was not specifically powered to detect statistically significant associations between dietary PUFA intake and neurodevelopmental outcomes. While the study provides valuable insights, studies designed specifically for this purpose would be needed to establish conclusive associations. Furthermore, a limitation of the study was the reliance on mean breast milk intake data from previous studies due to the unavailability of exact intake data. Despite these limitations, this study enhances our comprehension of PUFA intake in VLBW infants and sheds light on the potential impact on neurodevelopmental outcomes.

## 5. Conclusions

The findings of this study demonstrate that higher dietary intakes of total PUFAs, DHA and AA during the first year of life are associated with better neurodevelopmental outcome in terms of motor and cognitive function at 12 months CA. Additionally, the study highlights the need for improvement in dietary DHA and AA intakes during the CF period, as intakes were generally low and did not meet the recommended dietary intake levels. These results emphasize the importance of enhancing total PUFA, DHA and AA intake during CF as valuable goals, not only to comply with dietary guidelines but also to enhance neurological outcomes in very low birth weight (VLBW) infants. By providing evidence on the association between dietary PUFA intake and neurodevelopmental outcomes, this study contributes to our understanding of optimizing post-discharge nutritional management in this vulnerable population.

## Figures and Tables

**Table 1:** Main fatty acids of the *n*-3 and *n*-6 fatty acid families.

Fatty Acid Family	Number of Double Bonds	Name
*n*-3 fatty acids	18:3	alpha-Linolenic acid (ALA)
	22:6	Docosahexaenoic acid (DHA)
	20:5	Eicosapentaenoic acid (EPA)
*n*-6 fatty acids	18:2	Linoleic acid (LA)
	20:4	Arachidonic Acid (AA)

**Table 2:** Baseline characteristics of the PUFA subgroup—obstetric parameters and neonatal outcome.

Baseline Characteristics (*n* = 140)	
***Neonatal outcomes***	
Birth weight (g)	928 (719–1125)
Height at birth (cm)	35.0 (32.5–37.0)
Head circumference at birth (cm)	25 (23.0–26.3)
Gestational age (days)	192 (177–200)
Gestational age (weeks)	27/3
Time to full enteral feeds (days)	21 (14–31)
Male sex	79 (56%)
Intraventricular hemorrhage Grade III + IV	7 (5%)
Retinopathy of prematurity (any)	
Grade 1	11 (8%)
Grade 2	24 (17%)
Grade 3	6 (4%)
Nutrition at discharge	
Breastmilk	44 (31%)
Formula	44 (31%)
Mixed	52 (37%)
Necrotizing enterocolitis	
Grade 1	1 (0%)
Grade 2	2 (1%)
Periventricular leukomalacia	2 (1%)
***Obstetric and parental parameters***	
Age of mother at birth (years)	33 (30–37)
Multiple birth	43 (31%)
Cesarean delivery	129 (92%)
Preeclampsia	12 (9%)
Highest parental education	
Primary education	43 (31%)
Secondary education	23 (16%)
Tertiary education	58 (41%)
Maternal smoking habit	
Before pregnancy	27 (19%)
During pregnancy	4 (3%)
After pregnancy	3 (2%)
Always	19 (14%)

Categorical data are presented as numbers with percentages in parentheses. Continuous data are presented as median with interquartile range in parentheses. Parental education data were collected at study inclusion and divided into three groups according to highest education of either parent: primary education = compulsory school; secondary education = high school; tertiary education = postsecondary education. Necrotizing enterocolitis was diagnosed either clinically (Bell’s stage ≥ Ila) or after exploratory surgery. Cerebral ultrasound was used for the diagnosis of intraventricular hemorrhage and periventricular leukomalacia. Retinopathy of prematurity was diagnosed by direct ophthalmoscopy.

**Table 3:** Dietary total PUFA intake during complementary feeding and its association with neurodevelopmental outcome at 12 and 24 months CA.

Bayley-III	Bayley Assessment (Months CA)	*n*	ERS	95% CI	*p*-Value Unadjusted	*p*-Value Adjusted
Cognition	12	140	7.32	3.84 to 10.80	**0.0005**	**0.003**
	24	130	2.74	−1.71 to 7.18	0.20	0.31
Motor	12	140	4.73	1.80 to 7.67	**0.004**	**0.01**
	24	124	3.37	−0.24 to 6.97	0.06	0.13
Language	12	140	1.65	−1.46 to 4.75	0.27	0.32
	24	122	−0.61	−5.29 to 4.10	0.78	0.77

PUFA, polyunsaturated fatty acids; CA, corrected age; CI, confidence interval; ERS, estimated regression slope (increase in cognition/language/motor score per g PUFA/d). *p*-values < 0.05 are highlighted in bold.

**Table 4:** Dietary DHA intake during complementary feeding and its association with neurodevelopmental outcome at 12 and 24 months CA.

Bayley-III	Bayley Assessment (Months CA)	*n*	ERS	95% CI	*p*-Value Unadjusted	*p*-Value Adjusted
Cognition	12	140	0.16	0.00 to 0.32	**0.04**	0.13
	24	130	0.03	−0.16 to 0.23	0.75	0.77
Motor	12	140	0.21	0.10 to 0.34	**0.002**	**0.01**
	24	124	0.03	−0.13 to 0.19	0.68	0.77
Language	12	140	0.05	−0.08 to 0.18	0.42	0.77
	24	122	−0.03	−0.24 to 0.18	0.77	0.77

DHA, docosahexaenoic acid; CA, corrected age; CI, confidence interval; ERS, estimated regression slope (increase in cognition/language/motor score per mg DHA/d). *p*-values < 0.05 are highlighted in bold.

**Table 5:** Dietary AA intake during complementary feeding and its association with neurodevelopmental outcome at 12 and 24 months CA.

Bayley-III	Bayley Assessment (Months CA)	*n*	ERS	95% CI	*p*-Value Unadjusted	*p*-Value Adjusted
Cognition	12	140	0.11	−0.01 to 0.24	0.07	0.21
	24	130	−0.07	−0.22 to 0.07	0.32	0.48
Motor	12	140	0.16	0.06 to 0.26	**0.004**	**0.03**
	24	124	−0.07	−0.18 to 0.05	0.24	0.48
Language	12	140	0.02	−0.09 to 0.12	0.75	0.74
	24	122	−0.05	−0.22 to 0.12	0.53	0.63

AA, arachidonic acid; CA, corrected age; CI, confidence interval; ERS, estimated regression slope (increase in cognition/language/motor score per mg AA/d). *p*-values < 0.05 are highlighted in bold.

## Data Availability

The study protocol and the individual participant data that underlie the results reported in this article, after de-identification, are available upon request from the corresponding author 6 months after publication. Researchers will need to state the aims of any analyses and provide a methodologically sound proposal. Proposals should be directed to nadja.haiden@kepleruniklinikum.at. Data requestors will need to sign a data access agreement and in keeping with patient consent for secondary use, obtain ethical approval for any new analyses.

## Supplementary Materials

The following supporting information can be downloaded at: https://www.mdpi.com/article/10.3390/nu15143141/s1, Table S1: Baseline characteristic comparison between the intention to treat study population and the PUFA subgroup; Table S2: Baseline characteristic comparison between the PUFA subgroup and participants that were lost to follow up; Table S3: Dietary intake of total PUFAs, AA, DHA, linoleic acid, alpha-linolenic acid and n6:n3 ratio from 3 to 12 months CA. Table S4: Polyunsaturated Fatty Acid Intake and Neurological Outcome comparing sexes; Table S5: Docosahexaenoic Acid Intake and Neurological Outcome comparing sexes; Table S6: Arachidonic Acid Intake and Neurological Outcome comparing sexes.
