# Supplementary material for: Polyunsaturated Fatty Acid Intake during Complementary Feeding and Neurodevelopmental Outcome in Very Low Birth Weight Infants
Source: Nutrients. 2023 Jul 14;15(14):3141. doi: 10.3390/nu15143141 (PMC10385005; doi:10.3390/nu15143141)
Supplement: Supplementary file 1 [file nutrients-15-03141-s001.zip › nutrients-2481144-supplementary.pdf]

Supplemental Material

1) Baseline characteristics comparing the initial Intention-to-treat dataset with the PUFA subgroup

The PUFA subgroup is defined as all subjects in which at least one dietary record was valid for analysis and Bayley-III outcome at 12 months corrected age (CA) was available. Categorical data were tested using the $\chi 2$ test and are presented as numbers with percentages in round parentheses. Continuous data are presented as median with interquartile range in round parentheses and were tested using the Mann-Whitney U test. All tests were two-sided and p-values <0.05 were considered statistically significant.

Table S1. Baseline characteristic comparison between the Intention to treat study population and the PUFA subgroup.

**Baseline characteristics (n=140)**			
Neonatal outcome	***Intention to treat (n=177)***	***PUFA subgroup (n=140)***	***p-value***
Gestational age (days)	191 (177-201)	192 (177-200)	0.95
Birthweight (g)	930 (725-1140)	928 (719-1125)	0.81
Height at birth (cm)	35.0 (32.0-37.0)	35.0 (32.5-37.0)	0.82
Head circumference at birth (cm)	25 (23.0-26.5)	25 (23.0-26.3)	0.89
Time to full enteral feeds (days)	20 (14-39)	21 (14-31)	0.90
Male sex	98 (55%)	79 (56%)	0.94
Intraventricular hemorrhage grade III+IV	10 (6%)	7 (5%)	0.99
Anemia	18 (10%)	10 (7%)	0.45
Retinopathy of prematurity			
Grade 1	12 (7%)	11 (8%)	
Grade 2	30 (17%)	24 (17%)	0.81
Grade 3	10 (6%)	6 (4%)	
Perinatal steroids (full course)	104 (59%)	77 (55%)	0.46
Nutrition at discharge			
breastmilk	51 (29%)	44 (31%)	
formula	60 (34%)	44 (31%)	0.83
mixed	63 (36%)	52 (37%)	
Necrotizing Enterocolitis			
Grade 1	1 (0.6%)	1 (0.7%)	1.00
Grade 2	2 (1.1%)	2 (1.4%)	
Periventricular Leucomalacia	2 (1.1%)	2 (1.4%)	1.00
Obstetric and parental parameter			
Age mother at birth (years)	32 (29-37)	33 (30-37)	0.33
Multiple birth	60 (34%)	43 (31%)	0.61
Cesarean delivery	162 (92%)	129 (92%)	1.00
Praeclampsia	17 (10%)	12 (9%)	0.87
Highest parental education			
primary education	52 (29%)	43 (31%)	
secondary education	30 (17%)	23 (16%)	0.99
tertiary education	67 (38%)	58 (41%)	

Maternal smoking habit			
before pregnancy	33 (19%)	27 (19%)	
during pregnancy	4 (2%)	4 (3%)	0.99
after pregnancy	3 (2%)	3 (2%)	
always	23 (13%)	19 (14%)	

2) **Baseline characteristics comparing the PUFA subgroup and Infants that were lost to follow up**

Participants without dietary records and/or Bayley-III at 12 months CA were excluded from analysis (Lost to follow up). Categorical data were tested using the $\chi 2$ test and are presented as numbers with percentages in round parentheses. Continuous data are presented as median with interquartile range in round parentheses and were tested using the Mann-Whitney U test. All tests were two-sided and p-values <0.05 were considered statistically significant.

Table S2. Baseline characteristic comparison between the PUFA subgroup and participants that were lost to follow up .

**Baseline characteristics**			
Neonatal outcome	***PUFA subgroup (n=140)***	***Lost to follow up (n=37)***	***p-value***
Gestational age (days)	192 (177-200)	189 (177-202)	0.65
Birthweight (g)	928 (719-1125)	900 (740-1140)	0.71
Height at birth (cm)	35.0 (32.5-37.0)	35.0 (32.0-38.0)	0.72
Head circumference at birth (cm)	25 (23.0-26.3)	25 (22.8-26.7)	0.96
Time to full enteral feeds (days)	21.0 (14-31)	20.0 (15.5-24.75)	0.96
Male sex	79 (56%)	19 (52%)	0.58
Intraventricular hemorrhage grade III+IV	7 (5%)	3 (1%)	0.48
Anemia	10 (7%)	8 (22%)	0.03
Retinopathy of prematurity			
Grade 1	11 (8%)	1 (3%)	
Grade 2	24 (17%)	6 (16%)	0.23
Grade 3	6 (4%)	4 (11%)	
Perinatal steroids (full course)	77 (55%)	27 (73%)	0.01
Nutrition at discharge			
breastmilk	44 (31%)	7 (19%)	
formula	44 (31%)	16 (43%)	0.20
mixed	52 (37%)	11 (30%)	
Necrotizing Enterocolitis			
Grade 1	1 (0.7%)	0 (0%)	1.00
Grade 2	2 (1.4%)	0 (0%)	
Periventricular Leucomalacia	2 (1.4%)	0 (0%)	1.00
Obstetric and parental parameter			
Age mother at birth (years)	33 (30-37)	30 (24-33)	0.002
Multiple birth	43 (31%)	17 (46%)	0.12
Cesarean delivery	129 (92%)	33 (89%)	0.52

Praeclampsia	12 (9%)	4 (11%)	0.52
Highest parental education			
primary education	43 (31%)	10 (27%)	
secondary education	23 (16%)	7 (19%)	0.11
tertiary education	58 (41%)	8 (22%)	
Maternal smoking habit			
before pregnancy	27 (19%)	6 (16%)	
during pregnancy	4 (3%)	1 (3%)	1.00
after pregnancy	3 (2%)	0 (0%)	
always	19 (14%)	4 (11%)	

3) Fatty Acid Intake comparing Early and Late Introduction of Solid Foods

Nutrient intake was compared between early and late introduction of solid foods using linear mixed-effects models accounting for randomization group, sex, gestational age, and nutrition at discharge as covariates, with a random intercept to account for possible correlation between siblings of multiple births. Marginal means (i.e. averaged across covariates) for the two groups were calculated from the linear mixed models, together with according standard errors and p-values for the null hypothesis of no between-group difference. P values <0.05 were considered statistically significant.

Table S3. Dietary Intake of total PUFAs, AA, DHA, Linoleic acid, alpha-Linolenic acid and LA/ALA ratio from 3-12 months CA.

Nutrient	Total PUFA (g/d)					Docosahexaenoic Acid (mg/d)					Arachidonic Acid (mg/d)				
	Early		Late			Early		Late			Early		Late		
months CA	mean	SE	mean	SE	p-value	mean	SE	mean	SE	p-value	mean	IQR	mean	IQR	p-value
M3	4.3	0.1	4.3	0.1	0.97	73.0	2.7	78.4	2.8	0.17	87.1	4.2	93.7	4.3	0.29
M4	4.5	0.1	4.4	0.1	0.28	70.0	3.0	68.5	2.7	0.71	63.0	4.3	78.4	3.9	0.44
M5	4.6	0.2	4.6	0.2	0.90	67.4	3.0	68.8	3.1	0.73	78.2	4.0	78.7	4.0	0.93
M6	4.7	0.1	4.6	0.1	0.84	66.0	3.2	63.6	3.1	0.58	76.1	4.2	73.3	4.0	0.62
M7	4.5	0.2	4.7	0.1	0.43	56.3	3.5	61.3	3.2	0.30	66.3	4.4	66.6	4.0	0.95
M8	4.6	0.2	4.8	0.2	0.41	54.3	3.7	59.4	3.4	0.31	63.5	4.5	65.7	4.1	0.72
M9	4.9	0.2	4.8	0.2	0.60	56.9	3.2	54.9	3.1	0.66	64.6	3.9	61.6	3.7	0.57
M10	4.4	0.2	4.8	0.2	0.88	51.4	3.1	54.0	3.0	0.55	56.9	3.7	58.6	3.5	0.75
M11	4.6	0.2	4.9	0.2	0.24	52.6	4.5	52.0	4.1	0.92	55.0	5.9	58.4	5.4	0.68
M12	4.7	0.2	4.7	0.2	0.78	58.1	5.2	53.4	5.0	0.51	57.0	6.1	60.7	6.0	0.67
**nutrient**	**Linoleic acid (% of energy)**					**alpha-linolenic acid (% of energy)**					**LA/ALA Ratio**				
	**Early**		**Late**			**Early**		**Late**			**Early**		**Late**		
**months CA**	**mean**	**SE**	**mean**	**SE**	**p-value**	**mean**	**SE**	**mean**	**SE**	**p-value**	**mean**	**IQR**	**mean**	**IQR**	**p-value**
M3	6.34	0.13	6.38	0.13	0.84	0.80	0.03	0.83	0.03	0.69	8.24	0.26	8.34	0.28	0.78
M4	6.32	0.14	6.56	0.13	0.21	0.79	0.03	0.85	0.03	0.10	8.21	0.23	8.04	0.21	0.61
M5	6.01	0.13	6.12	0.13	0.23	0.77	0.02	0.83	0.02	0.07	7.95	0.19	7.80	0.20	0.57
M6	5.84	0.13	7.74	0.13	0.58	0.74	0.02	0.74	0.02	0.88	8.08	0.18	7.90	0.18	0.51
M7	5.42	0.14	5.60	0.13	0.38	0.68	0.03	0.70	0.02	0.59	8.22	0.27	8.21	0.24	0.97
M8	5.24	0.14	5.31	0.13	0.71	0.65	0.02	0.67	0.02	0.63	8.19	0.20	8.07	0.18	0.66
M9	5.16	0.13	5.23	0.13	0.74	0.66	0.02	0.66	0.02	0.88	8.07	0.23	8.02	0.22	0.88
M10	4.90	0.13	5.02	0.12	0.52	0.65	0.02	0.64	0.02	0.92	7.78	0.20	7.94	0.19	0.56
M11	4.62	0.16	4.93	0.14	0.17	0.61	0.02	0.63	0.02	0.59	7.67	0.26	8.07	0.25	0.31
M12	4.49	0.15	4.34	0.15	0.49	0.60	0.02	0.61	0.02	0.66	7.61	0.20	7.28	0.20	0.25

PUFA: Polyunsaturated fatty acids; LA: Linoleic acid; ALA: alpha-linolenic acid; SE: Standard Error

4) **Subgroup analysis – PUFA Intake and Neurological Outcome comparing sexes**

For the subgroup analysis to investigate the association of PUFAs with developmental neurological outcomes between sexes we used linear mixed-effects models with PUFA intake, gestational age at birth, intervention group, sex, highest parental education, nutrition at discharge (breastfed, formula, mixed (breastmilk and formula), and Intraventricular hemorrhage (Grades III+IV) as fixed effects, a random intercept to adjust for possible correlation between siblings of multiple births, and an interaction term for sex and PUFAs. Model estimates and 95% confidence intervals are reported.

Table S4. Polyunsaturated Fatty Acid Intake and Neurological Outcome comparing sexes.

Bayley-III	Bayley assessment (months CA)	sex	n	ERS	95% CI	p-value
**Cognition**	12	female	61	2.62	-2.58 to 7.81	**0.02**
		male	79	10.89	6.34 to 15.45	
	24	female	59	0.93	-5.79 to 7.66	0.45
		male	71	4.17	-1.91 to 10.25	
**Motor**	12	female	61	1.53	-2.92 to 5.98	0.06
		male	79	7.07	3.22 to 10.91	
	24	female	56	-0.88	-6.26 to 4.5	**0.04**
		male	68	6.83	1.94 to 11.7	
**Language**	12	female	61	-4.13	-8.72 to 0.465	**0.003**
		male	79	5.88	1.91 to 9.843	
	24	female	55	-4.97	-11.8 to 1.85	0.07
		male	67	3.05	-3.2 to 9.30	

PUFA: Polyunsaturated fatty acids, CA: corrected age, CI: Confidence Interval, ERS: Estimated Regression Slope (increase in cognition/language/motor score per g PUFA/d). p-values <0.05 are highlighted in bold.

Table S5. Docosahexaenoic Acid Intake and Neurological Outcome comparing sexes.

Bayley-III	Bayley assessment (months CA)	sex	n	ERS	95% CI	p-value
Cognition	12	female	61	-0.06	-0.29 to 0.17	**0.016**
		male	79	0.30	0.12 to 0.50	
	24	female	59	0.01	-0.29 to 0.31	0.82
		male	71	0.05	-0.21 to 0.30	
Motor	12	female	61	0.10	-0.09 to 0.29	0.09
		male	79	0.29	0.14 to 0.44	
	24	female	56	-0.04	-0.29 to 0.20	0.39
		male	68	0.08	-0.13 to 0.29	
Language	12	female	61	-0.14	-0.33 to 0.06	**0.02**
		male	79	0.17	0.01 to 0.33	
	24	female	55	-0.06	-0.37 to 0.25	0.76
		male	67	0.00	-0.29 to 0.28	

DHA: Docosahexaenoic acid, CA: corrected age, CI: Confidence Interval, ERS: Estimated Regression Slope (increase in cognition/language/motor score per g PUFA/d). p-values <0.05 are highlighted in bold.

Table S6. Arachidonic Acid Intake and Neurological Outcome comparing sexes.

Bayley-III	Bayley assessment (months CA)	sex	n	ERS	95% CI	p-value
Cognition	12	female	61	-0.04	-0.24 to 0.15	**0.04**
		male	79	0.02	0.05 to 0.35	
	24	female	59	-0.12	-0.35 to 0.11	0.54
		male	71	-0.04	-0.23 to 0.15	
Motor	12	female	61	1.09	-0.05 to 0.26	0.40
		male	79	1.84	0.06 to 0.30	
	24	female	56	-0.13	-0.31 to 0.06	0.36
		male	68	-0.03	-0.18 to 0.12	
Language	12	female	61	-0.08	-0.25 to 0.08	0.11
		male	79	0.07	-0.06 to 0.20	
	24	female	55	-0.07	-0.30 to 0.17	0.84
		male	67	-0.04	-0.26 to 0.18	

AA: Arachidonic acid, CA: corrected age, CI: Confidence Interval, ERS: Estimated Regression Slope (increase in cognition/language/motor score per g PUFA/d). p-values <0.05 are highlighted in bold.
